# Metabolic-Associated Steatotic Liver Disease: From Molecular Mechanisms to Novel Therapies

**DOI:** 10.1155/ijh/5580454

**Published:** 2025-06-23

**Authors:** John Cooper, Parth Patel, Joven Tristeza, Alexander Yang

**Affiliations:** Internal Medicine, University of Alabama, Birmingham, Alabama, USA

## Abstract

Metabolic-associated steatotic liver disease (MASLD) is a burgeoning worldwide burden and is currently the leading indication for a liver transplant. Despite the growing burden of disease, there are few pharmacological treatments available. The underlying molecular mechanisms of the development of MASLD are still being elucidated. In this review, we will summarize the known molecular mechanisms in the development of MASLD along with past, current, and future pharmacologic clinical trials.

## 1. Introduction

Metabolic-associated steatotic liver disease (MASLD), formerly known as nonalcoholic fatty liver disease (NALFD) [1], is a burgeoning worldwide burden affecting approximately a third of the worldwide population and is the leading indication for liver transplantation [2]. MASLD is a spectrum disease ranging from simple hepatic steatosis of the liver leading to steatohepatitis (MASH) which leads to progressive fibrosis and eventually cirrhosis [3]. This progression of disease is driven by a positive feedback loop of proinflammatory cytokines activating the innate immune system, leading to further increased inflammation [4]. MASH is an umbrella term that encompasses all metabolic-associated liver dysfunction, and recent studies have shown the heterogeneity of the disease, adding to the difficulty in conducting clinical studies [5]. As a result, there were no FDA-approved pharmaceutical treatments for MASH until recently.

Despite the new advancements in pharmaceutical treatments, the first-line treatment for MASLD remains lifestyle modification, including adhering to a diet that is low in saturated fats and high in polyunsaturated fatty acids such as the Mediterranean diet [6, 7]. On the contrary, a diet characterized by high saturated fatty acids and processed meats such as the Western diet has been shown to contribute to gut microbial dysbiosis, leading to the development of MASLD [8]. Furthermore, Ramadan fasting has also been shown to improve risk factors for MASLD, including hyperlipidemia, obesity, and diabetes [9]. The development and progression of MASLD are multifactorial and involve a combination of genetic susceptibility, metabolic, and oxidative stress, leading to the disruption of lipid homeostasis. This review will summarize the known mechanisms leading to the development of MASLD, along with both past, current, and future therapies to target these mechanisms.

## 2. Lipid Metabolism in the Development of MASLD

MASLD is defined as the presence of steatosis in at least 5% of hepatocytes either identified on imaging or histology in the absence of other causes of steatosis [3]. Fundamentally, the pathogenesis of MASLD is a perturbation of lipid homeostasis leading to excessive lipid accumulation in the liver. Thus, understanding the mechanisms of maintaining lipid homeostasis is vital for understanding the development of MASLD.

Excessive accumulation of free fatty acids (FFAs) in the plasma causes deleterious effects including insulin resistance, oxidative stress, and inflammation, a phenomenon known as “lipotoxicity” [10]. As a result, excessive FFAs are stored safely in the form of triacylglycerides (TAGs) on lipid droplets (LDs) located in the cytoplasm of the cell. The function of adipose tissue is to store TAGs for future energy expenditure and to act as a buffer against metabolic disease [11]. While adipose tissue has the ability to expand and accommodate, there is a limit to what can be stored in adipose tissue before TAG starts to accumulate in peripheral tissues, such as the liver, leading to the development of MASLD. This is best illustrated in patients with lipodystrophy who cannot form adipose tissue, leading to ectopic lipid accumulation resulting in hypertriglyceridemia, insulin resistance, and MASLD [12]. Similarly, patients with obesity consume excessive caloric intake, resulting in excessive FFAs stored in ectopic organs such as the liver, impairing insulin sensitivity [13]. Thus, both obesity and diabetes are well-known risk factors for MASLD.

## 3. The Relationship Between Type 2 Diabetes (T2DM) and MASLD

Mechanistically, insulin resistance impairs suppression of lipolysis from peripheral adipose tissue [14, 15]. This results in increased serum concentrations of FFAs and peripheral uptake in the liver, thus leading to increased TAG formation and the development of MASLD [16]. As a result of the contribution of insulin resistance to the development of MASLD, T2DM drugs that improve insulin resistance have been tested in the treatment of MASLD.

## 4. Effect of Antidiabetic Drugs on MASLD

Metformin is a popularly used antidiabetic medication in the biguanide subclass [17] that works by acting both directly and indirectly on the liver to decrease hepatic gluconeogenesis and increase glucose utilization [18]. On a biochemical level, metformin accumulates in hepatic mitochondria, inhibiting Complex I of the respiratory chain through the inhibition of ubiquinone reduction, thus decreasing hepatocellular ATP production [19, 20]. This increase in AMP:ATP and ADP:ATP ratios activates AMPK, which upregulates catabolic processes to restore energy homeostasis and downregulates catabolic activities that consume ATP, such as gluconeogenesis, improving insulin sensitivity [21].

Despite being first-line therapy for T2DM, the effects of metformin on the progression of MASLD are mixed at best. Studies in human models have shown varying results including decreasing transaminases (AST and ALT) in patients taking metformin but no significant decrease in hepatic steatosis or fibrosis via hepatic ultrasonography [22]. Patients undergoing metformin and n-acetylcysteine treatment for 12 months showed no significant change in hepatic transaminases (AST and ALT) but displayed statistically significant decrease in liver steatosis and fibrosis [23]. Further studies are warranted to determine metformin's effectiveness in the treatment and reversal of MASLD.

SGLT-2 inhibitors are a relatively new antidiabetic medication that lower serum glucose levels by decreasing reuptake of glucose through SGLT-2 transporters in the renal proximal tubule. These medications are a mainstay in the treatment of T2DM due to their ability to reduce bodyweight, serum glucose, and systolic blood pressure while providing both cardioprotective and nephroprotective properties [24–26]. The potential role of SGLT-2 inhibitors in the treatment of MASLD can be attributed to their ability to decrease hepatic TAGs and lipotoxic intermediates [27, 28]. A recent study also found that the SGLT-2 inhibitor empagliflozin ameliorated liver injury and lipid metabolism disorder by stimulating the AMPK/mTOR signaling pathway, thus enhancing macrophage autophagy in mice [29]. Although SGLT-2 inhibitors are not FDA-approved for the treatment of MASLD, recent studies have shown promising results for the use of SGLT-2 inhibitors in patients with MASLD.

In the E-LIFT trial, 50 patients with MASLD and diabetes were randomized to empagliflozin 10 mg daily versus placebo for 20 weeks. The empagliflozin group showed significant reduction of liver fat using MRI compared to placebo (−5.1%, *p* < 0.0001) and significant reduction of ALT (*p* = 0.005) [30]. In another randomized trial, 240 patients with both T2DM and MASLD were randomized to receive either empagliflozin 25 mg, ursodeoxycholic acid (UCDA), or placebo for 6 months. Empagliflozin significantly decreased liver fat content (LFC) (−8.73%, *p* < 0.0001) compared to placebo and showed significant regression in the FIB-4 index (−0.34, *p* = 0.011). More patients had normal fatty liver grade via ultrasound or MRI imaging when treated with empagliflozin compared to placebo [31]. However, in a meta-analysis that analyzed 212 patients, empagliflozin showed no improvement in liver stiffness measurement score, AST, or ALT levels compared to placebo [32]. Overall, more studies are needed to establish the efficacy of SGLT-2 inhibitors for the treatment of MASLD. This contrasts with SGLT-2 inhibitors in improving cardiovascular health, where they have shown a mortality benefit in those with heart failure through a variety of proposed mechanisms, including decreased inflammation, increased natriuresis, and improved cardiac energy metabolism [33].

Glucagon-like peptide-1 (GLP-1) receptor agonists have an established effect of body weight reduction by enhancing satiety through delayed gastric emptying and hypothalamic stimulation. They have been recently approved for obesity along with T2DM [34–36]. The weight loss associated with GLP-1 agonists has been shown to be associated with decreased hepatic inflammation in patients with MASH. The LEAN Phase II study showed that 39% (9/23) of participants who received liraglutide 1.8 mg SC daily had a resolution of MASH via liver biopsy compared with 9% (2/22) in the placebo group after the 48-week treatment interval. However, there was no significant improvement of liver fibrosis between the two groups [37]. This is being followed up by the ongoing Phase III multicenter ESSENCE trial that is evaluating once weekly semaglutide 2.4 mg compared to placebo in patients with biopsy-proven MASH and fibrosis Stage 2 or 3 planning to study the patients for 240 weeks [38]. In a late-breaking abstract to the 2024 The Liver Meeting, initial data showed that upon analysis of 800 participants in the study, semaglutide showed significant resolution of steatohepatitis with no worsening of fibrosis compared to placebo (62.9% vs. 34.1%, *p* < 0.001) along with the improvement of liver fibrosis with no worsening of steatohepatitis (37% vs. 22.5%, *p* < 0.001) [39]. Although these data are promising, the trial is still ongoing, and GLP-1 agonists are not FDA-approved for the treatment of MASH.

There is an emerging evidence that a new GLP-1/glucagon receptor coagonist, efinopegdutide, might lead to greater reduction in LFC than GLP-1 agonists. A Phase IIa, randomized, active-comparator-controlled, parallel-group, open-label study of 145 patients with MASLD showed that efinopegdutide 10 mg weekly led to a greater relative reduction in LFC than semaglutide (72.7% vs. 42.3%, *p* < 0.001) after 24 weeks of therapy while leading to a statistically insignificant reduction in baseline body weight (efinopegdutide 8.5% vs. semaglutide 7.1%; *p* = 0.085) [40]. While GLP-1 agonists reduce body weight and LFC through extrahepatic mechanisms, it is postulated that efinopegdutide's additional LFC reduction can be attributed to the glucagon activation of fatty acid oxidation. The results of this study suggest that efinopegdutide and other GLP-1 agonists/glucagon receptor coagonists could be utilized as a novel therapy for MAFLD by reducing LFC, therefore decreasing progression of fibrosis [41].

## 5. The Role of PPAR Receptors in Lipid Metabolism

The peroxisome proliferator-activated receptors (PPAR-*α*, PPAR-*β*/delta, and PPAR-*γ*) are members of the nuclear receptor family of ligand-dependent transcription factors that regulate lipid metabolism [42, 43]. FFAs are the main ligands for PPAR, and the activation of this class of receptors results in the downstream expression of their target genes responsible for maintaining lipid homeostasis throughout the entire body [44, 45].

PPAR-*α* is the most abundant PPAR isotype found in the liver and regulates the genes mediating FFA transportation, hepatic inflammation, *β*-oxidation, and lipoprotein regulation [46–48]. Mice deficient in hepatocyte-specific PPAR-*α* display impaired FA oxidation and spontaneously develop hepatic steatosis in the liver [49]. In addition, the activation of PPAR-*α* has also been shown to inhibit the activation of hepatic stellate cells (HSCs) which improves liver fibrosis [50].

On the other hand, PPAR-*γ* is mostly expressed in adipocytes. The activation of PPAR-*γ* results in adipogenesis and the expansion of adiposity [51]. PPAR-*γ* mutations in humans have been shown to cause familial lipodystrophy [52]. Rosiglitazone and pioglitazone, known as thiazolidinediones (TZDs), are PPAR-*γ* agonists and are approved for the treatment of T2DM. However, the increased risk of cardiovascular mortality, weight gain, and fractures has caused PPAR *γ* agonists to be taken off the European market [53, 54]. Taken together, both PPAR *α* and *γ* receptors could be viable targets for the treatment of MASLD.

## 6. Targeting PPAR Receptors for the Treatment of MASLD

Given the beneficial effects of PPAR-*γ* on enhancing adiposity, PPAR-*γ* agonist drugs have been tested in clinical trials for MASLD. In the landmark PIVENS trial, 247 patients with MASH without diabetes were randomized to receive pioglitazone, vitamin E, or placebo for 96 weeks. Treatment of pioglitazone led to significant reductions in liver transaminases (*p* < 0.001) and lobular inflammation (*p* = 0.004) compared to placebo but no improvement in fibrosis scores (*p* = 0.12) [55].

Similarly, in the FLIRT trial, 63 patients with biopsy-proven MASH were randomized to receive rosiglitazone or placebo for a year. Patients treated with rosiglitazone were significantly more likely to have improvement in hepatic steatosis (47% vs. 16%, *p* = 0.014) and normalized transaminase levels (38% vs. 7%, *p* = 0.005). However, there were no improvement in histologic legions including fibrosis, or MASLD activity score [56]. After a year, the same patients were enrolled in an open-label 2-year extension of the trial named FLIRT2. Despite the extension, the treatment of rosiglitazone still did not lead to a significant change in MASH activity score, ballooning score, or fibrosis stage despite maintaining effects on increasing insulin sensitivity and restoring transaminase levels [57].

Like PPAR-*γ* agonists, PPAR-*α* agonists have shown mixed results when tested for the treatment of MASLD. In a randomized, double-blinded, placebo-controlled study named the EFFECT1 trial, 78 patients were randomized to receive omega-3 carboxylic acid, fenofibrate, or placebo for 12 weeks. Although fenofibrate reduced serum TAG levels, fenofibrate actually lead to significantly increased liver volumes and total liver fat as detected by MRI [58].

Saroglitazar, a dual agonist of PPAR-*γ* and PPAR-*α*, was the first international drug approved for the treatment of MASLD in India [59]. In a series of trials named EVIDENCES, the pharmacological company Zydus tested saroglitazar for the treatment of MASH. In the proof-of-concept study EVIDENCES IV, 16 adults with biopsy-proven MASH were randomized to placebo, saroglitazar 2 mg, or saroglitazar 4 mg for 24 weeks. Significant improvement in hepatocyte ballooning, steatosis, and resolution of steatohepatitis was seen in both treatment groups compared to placebo [60]. This was followed by Phase III trials, where saroglitazar was shown to significantly decrease LFC and liver stiffness compared to placebo after 24 weeks of treatment. Zydus is enrolling 1500 MASH patients in a Phase IV trial named EVIDENCES XI. In this study, they plan to measure liver stiffness changes as measured by transient elastography after 52 weeks of treatment with saroglitazar [61]. This ambitious study will be one of the largest prospective studies for the treatment of MASH.

## 7. The Role of Thyroid Hormone in Maintaining Lipid Homeostasis

Thyroid hormones are essential for maintaining metabolic homeostasis in the entire body including the liver, where they regulate lipid metabolism. There are two isoforms of thyroid hormone receptors, THR-*α* and THR-*β*, with THR-*β* being the predominant form in the liver [62, 63]. Activation of THR-*β* results in increased mobilization of FFAs from stored TAGs and increases beta-oxidation of these released FFAs, thus overall resulting in a net negative effect in total hepatic triglycerides [64, 65]. Patients with untreated hypothyroidism have increased blood concentrations of TAGs and have an increased risk for developing MASLD [66–69]. Taken together, thyroid hormone receptors in the liver represent a potential therapeutic target for MASLD.

## 8. Targeting Hepatic Specific Thyroid Receptor in MASLD

The selective targeting and activation of THR-*β* in the liver has shown to reduce hepatic fat and atherogenic lipids such as LDL, triglyceride, and apolipoprotein B without triggering excessive thyroid hormone stimulation in other organs, thus leading to the development of resmetirom, a liver-specific THR-*β* agonist [70]. In a randomized Phase 3 trial involving patients with biopsy-proven MASH and fibrosis from Stages F1B–F3, 966 patients were randomized to once daily dose of 80 or 100 mg of resmetirom or placebo (1:1:1). Both doses of resmetirom significantly improved fibrosis by at least one stage of fibrosis (*p* < 0.001 for both comparisons with placebo) [71]. As a result of this Phase 3 trial, resmetirom has become the first FDA-approved drug for MASLD here in the United States.

## 9. Reactive Oxygen Species (ROS) and the Development of MASLD

ROS are produced as byproducts of metabolism predominantly in the mitochondria of the cell. When ROS are generated, antioxidant scavenger systems neutralize the ROS, thus maintaining cellular homeostasis. However, in the development of MASLD, hepatic lipid overload results in the impairment of the electron transport chain and increased formation of ROS. The ROS react with macromolecules such as lipids, forming highly unstable lipid hydroperoxides, increasing oxidative stress within the cell. Vitamin E is an antioxidant that can buffer the deleterious effects of ROS and thus has been implicated as a potential therapeutic option for MASLD [72, 73].

In the landmark PIVENS trial, nondiabetic patients with MASH who received vitamin E (800 IU/day) had improved liver enzymes, steatosis, lobular inflammation, and hepatocyte ballooning compared to placebo. However, no significant improvement in liver fibrosis was noted. The use of vitamin E was associated with adverse effects, however, including increased all-cause mortality and hemorrhagic stroke [55]. As a result, the AASLD only recommends vitamin E for biopsy-proven MASH after carefully discussing the risks and benefits with each patient before starting therapy.

## 10. The Role of Patatin-Like Phospholipase Domain-Containing Protein 3 (PNPLA3) in the Development of MASLD

The genetic component of MASLD was largely revealed in the 2008 landmark genome-wide association of the Dallas Heart Study cohort by Romeo et al., which identified significant association of the Ile148Met (I148M) variant of PNPLA3 that was associated with MASLD [74]. Since this study, the PNPLA3 I148M variant has been shown to be causal for the development of MASLD independent of BMI or diabetes [75]. The frequency of the allele is highest in Hispanics, with approximately 49% allele frequency. As a result, PNPLA3 remains a highly coveted potential personalized therapeutic target for the treatment of MASLD.

## 11. PNPLA3 as a Therapeutic Target for MASLD

Despite the prevalence of the I148M variant, the role of PNPLA3 and its interplay with the I148M variant in the development of MASLD remains controversial. Initially, recombinant human PNPLA3 displayed lipase activity against TAG, and the I148M variant had decreased TAG activity, leading to a loss of function theory [76]. However, this was soon refuted as the PNPLA3 KO mice did not develop hepatic steatosis [77]. Further studies showed that PNPLA3 I148M abnormally accumulates on the LDs due to decreased proteasome degradation. This phenomenon precipitated the development of MASLD in mice, suggesting a gain of function mutation [78]. Furthermore, PNPLA3 I148M was shown to suppress lipolysis by competing with adipose triglyceride lipase, suggesting a potential mechanism of how the I148M variant disrupts lipid homeostasis in the liver [79]. Antisense oligonucleotide (ASO)–mediated silencing of PNPLA3 in mice overexpressing the human PNPLA3 I148M reduced liver inflammation and fibrosis [80]. Recent Phase I trials of AZD2693, a potent PNPLA3 ASO, displayed knockdown of PNPLA3 and significant reduction of hepatic steatosis in patients carrying the PNPLA3 I148M allele and presumed MASH with an acceptable safety and tolerability profile [81]. Ongoing Phase II and III trials will hopefully result in FDA approval of PNPLA3 ASO in MASLD patients carrying the PNPLA3 I148M allele.

## 12. Conclusion

**Figure 1:**
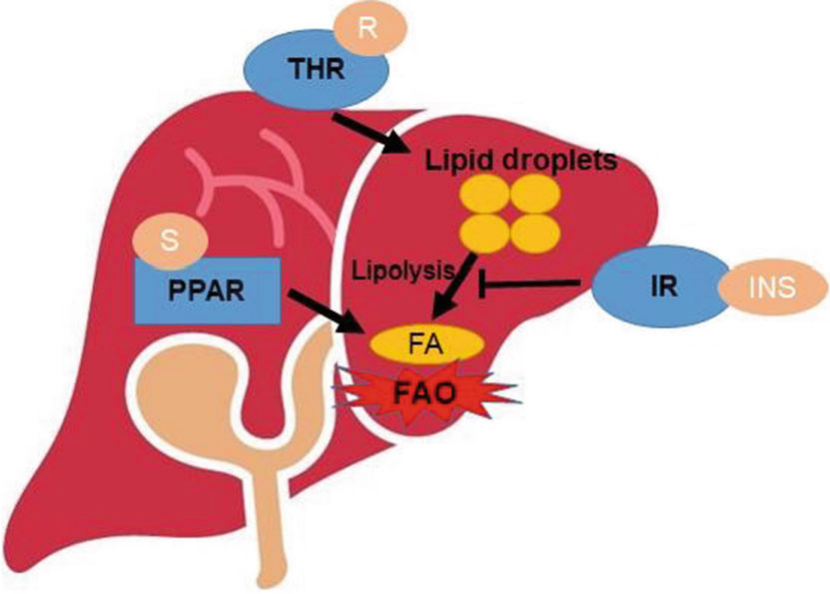
Summary of the mechanisms of key drugs for MASLD. THR = thyroid receptor, R = resmetirom, S = saroglitazar, IR = insulin receptor, INS = insulin, FAO = fatty acid oxidation. Resmetirom binding to the THR leads to increased lipolysis, while saroglitazar binding to PPAR receptors increases FAO. Both mechanisms lead to decreased TAG content in the liver. In the fed state, insulin suppresses lipolysis, and diabetic drugs increase insulin sensitivity.

While the incidence and prevalence of MASLD continue to grow significantly internationally, there are few pharmacological therapeutic options available. A summary of the key drugs and studies mentioned in our review paper is shown in Table 1, and the mechanisms of the drugs are summarized in Figure 1. Despite the lack of pharmacological therapeutic options, the future for the treatment of MASLD remains bright as new emerging therapeutic targets are being identified. With the expansion of genomic testing, MASLD is a tantalizing target for precision medicine as causal genomic variants have been identified. Overall, the field is highly dynamic, and with new therapeutic options on the horizon, the incidence of MASLD will hopefully decrease globally in the next coming years.

## Figures and Tables

**Table 1:** Summary of the classes of drugs for MASLD and the key clinical trials with results.

**Class of drugs**	**Mechanism of action**	**Individual drug names**	**Trial name and reference**	**Number of participants, length of study**	**Key findings**
PPAR-*γ* agonists	Binds to PPAR-*γ* receptors increasing adipogenesis	Rosiglitazone	FLIRT2 (Ratziu, 2010)	63 patients, 52 weeks	Pioglitazone improved steatosis and transaminases but no improvement in fibrosis
			PIVENS (Sanyal, 2010)	247 patients, 96 weeks	Pioglitazone associated with reduction in hepatic steatosis, ALT/AST levels, but no improvement in fibrosis score
			FLIRT (Ratziu, 2008)	53 patients, 3 years	Additional 2 years of pioglitazone did not show improvement in fibrosis, ballooning, or NAS score

PPAR-*α* agonists	Binds to PPAR-*α* receptors increasing FA oxidation	Fenofibrate	EFFECT 1 (Oscarsson, 2018)	78 patients, 12 weeks	Fenofibrate reduced serum triglycerides but increased total liver fat and volume

Dual PPAR *α*/*γ* agonists	Binds to both PPAR-*α*/*γ* receptors	Saroglitazar	EVIDENCES IV (Naga, 2021)	106 patients, 16 weeks	Saroglitazar reduced ALT levels and liver fat content assessed by MRI
		Elafibranor	GOLDEN505 (Sanyal, 2016)	276 patients, 52 weeks	Elafibranor resolved MASH in larger proportion compared to placebo in post hoc analysis. Intention to treat was insignificant

Thyroid hormone receptor agonists	Binds to liver-specific thyroid receptor *β*	Resmetirom	MAESTRO-NASH (Labriola, 2024)	966 patients, 52 weeks	Resmetirom significantly improved fibrosis and resolved NASH compared to placebo

Antidiabetic drugs	Inhibiting gluconeogenesis, activating AMPK	Metformin	Dagalp, 2004	36 patients, 24 weeks	Metformin did not have significant effect on fibrosis compared to placebo
			Hoofnagle, 2009	18 patients, 48 weeks	Metformin significantly reduced weight and improved MASH activity compared to placebo
			Merat, 2012	33 patients, 24 weeks	Compared to placebo (weight loss), metformin did not have significant effect on liver enzymes
	Inhibits SGLT-2 reuptake of glucose leading to AMPK activation	Empagliflozin	Rabea, 2022	240 patients, 24 weeks	Empagliflozin significantly reduced FIB-4 score and fatty liver grade on ultrasound
			E-LIFT (Kuchay, 2018)	50 patients, 20 weeks	Empagliflozin reduced liver fat as measured on MRI and decreased ALT compared to placebo
	GLP-1 agonist leading to satiety and weight loss	Semaglutide	NN9931-4296 (Harrison, 2021)	320 patients, 72 weeks	Semaglutide significantly resolved MASH without improving fibrosis

## Data Availability

Data sharing is not applicable to this article as no new data were created or analyzed in this study.

## References

[1] Rinella M. E., Lazarus J. V., Ratziu V. (2023). A Multisociety Delphi Consensus Statement on New Fatty Liver Disease Nomenclature. *Hepatology*.

[2] Younossi Z., Anstee Q. M., Marietti M. (2018). Global Burden of NAFLD and NASH: Trends, Predictions, Risk Factors and Prevention. *Nature Reviews Gastroenterology & Hepatology*.

[3] Saiman Y., Duarte-Rojo A., Rinella M. E. (2022). Fatty Liver Disease: Diagnosis and Stratification. *Annual Review of Medicine*.

[4] Kubes P., Mehal W. Z. (2012). Sterile Inflammation in the Liver. *Gastroenterology*.

[5] Luukkonen P. K. (2025). Subtypes of MASLD Confer Distinct Clinical Trajectories. *Journal of Hepatology*.

[6] Anania C., Perla F. M., Olivero F., Pacifico L., Chiesa C. (2018). Mediterranean Diet and Nonalcoholic Fatty Liver Disease. *World Journal of Gastroenterology*.

[7] Mourouti N., Kontogianni M. D., Papavagelis C. (2014). Adherence to the Mediterranean Diet Is Associated With Lower Likelihood of Breast Cancer: A Case-Control Study. *Nutrition and Cancer*.

[8] Jiménez-González C., Alonso-Peña M., Argos Vélez P., Crespo J., Iruzubieta P. (2025). Unraveling MASLD: The Role of Gut Microbiota, Dietary Modulation, and AI-Driven Lifestyle Interventions. *Nutrients*.

[9] Ahmed M., Ahmed M. H. (2024). Ramadan Fasting in Individuals With Metabolic Dysfunction-Associated Steatotic Liver Disease, Liver Transplant, and Bariatric Surgery: A Narrative Review. *Journal of Clinical Medicine*.

[10] Lee Y., Hirose H., Ohneda M., Johnson J. H., McGarry J. D., Unger R. H. (1994). Beta-Cell Lipotoxicity in the Pathogenesis of Non-Insulin-Dependent Diabetes Mellitus of Obese Rats: Impairment in Adipocyte-Beta-Cell Relationships. *Proceedings of the National Academy of Sciences of the United States of America*.

[11] Wronska A., Kmiec Z. (2012). Structural and Biochemical Characteristics of Various White Adipose Tissue Depots. *Acta Physiologica*.

[12] Fiorenza C. G., Chou S. H., Mantzoros C. S. (2011). Lipodystrophy: Pathophysiology and Advances in Treatment. *Nature Reviews Endocrinology*.

[13] Engin A. B. (2024). Mechanism of Obesity-Related Lipotoxicity and Clinical Perspective. *Advances in Experimental Medicine and Biology*.

[14] Girousse A., Tavernier G., Valle C. (2013). Partial Inhibition of Adipose Tissue Lipolysis Improves Glucose Metabolism and Insulin Sensitivity Without Alteration of Fat Mass. *PLoS Biology*.

[15] Samuel V. T., Petersen K. F., Shulman G. I. (2010). Lipid-Induced Insulin Resistance: Unravelling the Mechanism. *Lancet*.

[16] Donnelly K. L., Smith C. I., Schwarzenberg S. J., Jessurun J., Boldt M. D., Parks E. J. (2005). Sources of Fatty Acids Stored in Liver and Secreted via Lipoproteins in Patients With Nonalcoholic Fatty Liver Disease. *Journal of Clinical Investigation*.

[17] Bailey C. J., Day C. (1989). Traditional Plant Medicines as Treatments for Diabetes. *Diabetes Care*.

[18] Kim Y. D., Park K. G., Lee Y. S. (2008). Metformin Inhibits Hepatic Gluconeogenesis Through AMP-Activated Protein Kinase-Dependent Regulation of the Orphan Nuclear Receptor SHP. *Diabetes*.

[19] Zhang C. S., Li M., Ma T. (2016). Metformin Activates AMPK Through the Lysosomal Pathway. *Cell Metabolism*.

[20] Bridges H. R., Jones A. J., Pollak M. N., Hirst J. (2014). Effects of Metformin and Other Biguanides on Oxidative Phosphorylation in Mitochondria. *Biochemical Journal*.

[21] Hawley S. A., Ross F. A., Chevtzoff C. (2010). Use of Cells Expressing Gamma Subunit Variants to Identify Diverse Mechanisms of AMPK Activation. *Cell Metabolism*.

[22] Uygun A., Kadayifci A., Isik A. T. (2004). Metformin in the Treatment of Patients With Non-Alcoholic Steatohepatitis. *Alimentary Pharmacology & Therapeutics*.

[23] de Oliveira C. P., Stefano J. T., de Siqueira E. R. (2008). Combination of *N*-Acetylcysteine and Metformin Improves Histological Steatosis and Fibrosis in Patients With Non-Alcoholic Steatohepatitis. *Hepatology Research*.

[24] Shubrook J. H., Bokaie B. B., Adkins S. E. (2015). Empagliflozin in the Treatment of Type 2 Diabetes: Evidence to Date. *Drug Design, Development and Therapy*.

[25] Desouza C. V., Gupta N., Patel A. (2015). Cardiometabolic Effects of a New Class of Antidiabetic Agents. *Clinical Therapeutics*.

[26] Fonseca-Correa J. I., Correa-Rotter R. (2021). Sodium-Glucose Cotransporter 2 Inhibitors Mechanisms of Action: A Review. *Frontiers in Medicine*.

[27] Hüttl M., Markova I., Miklankova D. (2021). In a Prediabetic Model, Empagliflozin Improves Hepatic Lipid Metabolism Independently of Obesity and Before Onset of Hyperglycemia. *International Journal of Molecular Sciences*.

[28] Androutsakos T., Nasiri-Ansari N., Bakasis A. D. (2022). SGLT-2 Inhibitors in NAFLD: Expanding Their Role Beyond Diabetes and Cardioprotection. *International Journal of Molecular Sciences*.

[29] Meng Z., Liu X., Li T. (2021). The SGLT2 Inhibitor Empagliflozin Negatively Regulates IL-17/IL-23 Axis-Mediated Inflammatory Responses in T2DM With NAFLD via the AMPK/mTOR/Autophagy Pathway. *International Immunopharmacology*.

[30] Kuchay M. S., Krishan S., Mishra S. K. (2018). Effect of Empagliflozin on Liver Fat in Patients With Type 2 Diabetes and Nonalcoholic Fatty Liver Disease: A Randomized Controlled Trial (E-LIFT Trial). *Diabetes Care*.

[31] Elhini S. H., Wahsh E. A., Elberry A. A. (2022). The Impact of an SGLT2 Inhibitor Versus Ursodeoxycholic Acid on Liver Steatosis in Diabetic Patients. *Pharmaceuticals (Basel)*.

[32] Tang X., Zhang H., Wang X., Yang D. (2022). Empagliflozin for the Treatment of Non-Alcoholic Fatty Liver Disease: A Meta-Analysis of Randomized Controlled Trials. *African Health Sciences*.

[33] Lopaschuk G. D., Verma S. (2020). Mechanisms of Cardiovascular Benefits of Sodium Glucose Co-Transporter 2 (SGLT2) Inhibitors: A State-of-the-Art Review. *JACC: Basic to Translational Science*.

[34] Coveleskie K., Kilpatrick L. A., Gupta A. (2017). The Effect of the GLP-1 Analogue Exenatide on Functional Connectivity Within an NTS-Based Network in Women With and Without Obesity. *Obesity Science & Practice*.

[35] Nauck M. A., Meier J. J. (2018). Incretin Hormones: Their Role in Health and Disease. *Diabetes, Obesity & Metabolism*.

[36] Ard J., Fitch A., Fruh S., Herman L. (2021). Weight Loss and Maintenance Related to the Mechanism of Action of Glucagon-Like Peptide 1 Receptor Agonists. *Advances in Therapy*.

[37] Loomba R., Abdelmalek M. F., Armstrong M. J. (2023). Semaglutide 2.4 mg Once Weekly in Patients With Non-Alcoholic Steatohepatitis-Related Cirrhosis: A Randomised, Placebo-Controlled Phase 2 Trial. *Lancet Gastroenterology & Hepatology*.

[38] Newsome P. N., Sanyal A. J., Engebretsen K. A. (2024). Semaglutide 2.4 mg in Participants With Metabolic Dysfunction-Associated Steatohepatitis: Baseline Characteristics and Design of the Phase 3 ESSENCE Trial. *Alimentary Pharmacology & Therapeutics*.

[39] Alkhouri N. (2024). Phase 3 ESSENCE Trial: Semaglutide in Metabolic Dysfunction-Associated Steatohepatitis. *Gastroenterology & Hepatology (N Y)*.

[40] Romero-Gómez M., Lawitz E., Shankar R. R. (2023). A Phase IIa Active-Comparator-Controlled Study to Evaluate the Efficacy and Safety of Efinopegdutide in Patients With Non-Alcoholic Fatty Liver Disease. *Journal of Hepatology*.

[41] Boland M. L., Laker R. C., Mather K. (2020). Resolution of NASH and Hepatic Fibrosis by the GLP-1R/GcgR Dual-Agonist Cotadutide via Modulating Mitochondrial Function and Lipogenesis. *Nature Metabolism*.

[42] Mirza A. Z., Althagafi I. I., Shamshad H. (2019). Role of PPAR Receptor in Different Diseases and Their Ligands: Physiological Importance and Clinical Implications. *European Journal of Medicinal Chemistry*.

[43] Lee C. H., Olson P., Evans R. M. (2003). Minireview: Lipid Metabolism, Metabolic Diseases, and Peroxisome Proliferator-Activated Receptors. *Endocrinology*.

[44] Harmon G. S., Lam M. T., Glass C. K. (2011). PPARs and Lipid Ligands in Inflammation and Metabolism. *Chemical Reviews*.

[45] Mottillo E. P., Bloch A. E., Leff T., Granneman J. G. (2012). Lipolytic Products Activate Peroxisome Proliferator-Activated Receptor (PPAR) *α* and *δ* in Brown Adipocytes to Match Fatty Acid Oxidation With Supply. *Journal of Biological Chemistry*.

[46] Kroker A. J., Bruning J. B. (2015). Review of the Structural and Dynamic Mechanisms of PPAR*γ* Partial Agonism. *PPAR Research*.

[47] Szychowski K. A., Leja M. L., Kaminskyy D. V. (2017). Anticancer Properties of 4-Thiazolidinone Derivatives Depend on Peroxisome Proliferator-Activated Receptor Gamma (PPAR*γ*). *European Journal of Medicinal Chemistry*.

[48] Lin Y., Wang Y., Li P. F. (2022). PPAR*α*: An Emerging Target of Metabolic Syndrome, Neurodegenerative and Cardiovascular Diseases. *Frontiers in Endocrinology*.

[49] Bougarne N., Weyers B., Desmet S. J. (2018). Molecular Actions of PPAR*α* in Lipid Metabolism and Inflammation. *Endocrine Reviews*.

[50] Ip E., Farrell G., Hall P., Robertson G., Leclercq I. (2004). Administration of the Potent PPARalpha Agonist, Wy-14,643, Reverses Nutritional Fibrosis and Steatohepatitis in Mice. *Hepatology*.

[51] Towfighi A., Ovbiagele B. (2008). Partial Peroxisome Proliferator-Activated Receptor Agonist Angiotensin Receptor Blockers. Potential Multipronged Strategy in Stroke Prevention. *Cerebrovascular Diseases*.

[52] Campeau P. M., Astapova O., Martins R. (2012). Clinical and Molecular Characterization of a Severe Form of Partial Lipodystrophy Expanding the Phenotype of PPAR*γ* Deficiency. *Journal of Lipid Research*.

[53] Liu J., Wang L. N. (2023). Peroxisome Proliferator-Activated Receptor Gamma Agonists for Preventing Recurrent Stroke and Other Vascular Events in People With Stroke or Transient Ischaemic Attack. *Cochrane Database of Systematic Reviews*.

[54] Villacorta L., Schopfer F. J., Zhang J., Freeman B. A., Chen Y. E. (2009). PPARgamma and Its Ligands: Therapeutic Implications in Cardiovascular Disease. *Clinical Science*.

[55] Sanyal A. J., Chalasani N., Kowdley K. V. (2010). Pioglitazone, Vitamin E, or Placebo for Nonalcoholic Steatohepatitis. *New England Journal of Medicine*.

[56] Ratziu V., Giral P., Jacqueminet S. (2008). Rosiglitazone for Nonalcoholic Steatohepatitis: One-Year Results of the Randomized Placebo-Controlled Fatty Liver Improvement With Rosiglitazone Therapy (FLIRT) Trial. *Gastroenterology*.

[57] Ratziu V., Charlotte F., Bernhardt C. (2010). Long-Term Efficacy of Rosiglitazone in Nonalcoholic Steatohepatitis: Results of the Fatty Liver Improvement by Rosiglitazone Therapy (FLIRT 2) Extension Trial. *Hepatology*.

[58] Oscarsson J., Önnerhag K., Risérus U. (2018). Effects of Free Omega-3 Carboxylic Acids and Fenofibrate on Liver Fat Content in Patients With Hypertriglyceridemia and Non-Alcoholic Fatty Liver Disease: A Double-Blind, Randomized, Placebo-Controlled Study. *Journal of Clinical Lipidology*.

[59] Zydus (2020). *Zydus Announces the Approval of Saroglitazar Mg for the Treatment of Non-Alcoholic Fatty Liver Disease in India*.

[60] Goyal O., Nohria S., Goyal P. (2020). Saroglitazar in Patients With Non-Alcoholic Fatty Liver Disease and Diabetic Dyslipidemia: A Prospective, Observational, Real World Study. *Scientific Reports*.

[61] Zydus (2023). *Zydus Announces Phase IV EVIDENCES-XI Trial to Generate Real World Evidence of Saroglitazar in NAFLD Patients With Comorbidities*.

[62] Chamba A., Neuberger J., Strain A., Hopkins J., Sheppard M. C., Franklyn J. A. (1996). Expression and Function of Thyroid Hormone Receptor Variants in Normal and Chronically Diseased Human Liver. *Journal of Clinical Endocrinology & Metabolism*.

[63] Lazar M. A. (1993). Thyroid Hormone Receptors: Multiple Forms, Multiple Possibilities. *Endocrine Reviews*.

[64] Cable E. E., Finn P. D., Stebbins J. W. (2009). Reduction of Hepatic Steatosis in Rats and Mice After Treatment With a Liver-Targeted Thyroid Hormone Receptor Agonist. *Hepatology*.

[65] Erion M. D., Cable E. E., Ito B. R. (2007). Targeting Thyroid Hormone Receptor-Beta Agonists to the Liver Reduces Cholesterol and Triglycerides and Improves the Therapeutic Index. *Proceedings of the National Academy of Sciences of the United States of America*.

[66] Chung G. E., Kim D., Kim W. (2012). Non-Alcoholic Fatty Liver Disease Across the Spectrum of Hypothyroidism. *Journal of Hepatology*.

[67] Guo Z., Li M., Han B., Qi X. (2018). Association of Non-Alcoholic Fatty Liver Disease With Thyroid Function: A Systematic Review and Meta-Analysis. *Digestive and Liver Disease*.

[68] Kowalik M. A., Columbano A., Perra A. (2018). Thyroid Hormones, Thyromimetics and Their Metabolites in the Treatment of Liver Disease. *Frontiers in Endocrinology (Lausanne)*.

[69] Mandato C., D'Acunzo I., Vajro P. (2018). Thyroid Dysfunction and Its Role as a Risk Factor for Non-Alcoholic Fatty Liver Disease: What's New. *Digestive and Liver Disease*.

[70] Karim G., Bansal M. B. (2023). Resmetirom: An Orally Administered, Smallmolecule, Liver-Directed, *β*-Selective THR Agonist for the Treatment of Non-Alcoholic Fatty Liver Disease and Non-Alcoholic Steatohepatitis. *touchREVIEWS in Endocrinology*.

[71] Harrison S. A., Taub R. (2024). A Phase 3 Trial of Resmetirom in NASH With Liver Fibrosis. Reply. *New England Journal of Medicine*.

[72] Masarone M., Rosato V., Dallio M. (2018). Role of Oxidative Stress in Pathophysiology of Nonalcoholic Fatty Liver Disease. *Oxidative Medicine and Cellular Longevity*.

[73] Palmieri V. O., Grattagliano I., Portincasa P., Palasciano G. (2006). Systemic Oxidative Alterations Are Associated With Visceral Adiposity and Liver Steatosis in Patients With Metabolic Syndrome. *Journal of Nutrition*.

[74] Romeo S., Kozlitina J., Xing C. (2008). Genetic Variation in *PNPLA3* Confers Susceptibility to Nonalcoholic Fatty Liver Disease. *Nature Genetics*.

[75] Li J. Z., Huang Y., Karaman R. (2012). Chronic Overexpression of PNPLA3^I148M^ in Mouse Liver Causes Hepatic Steatosis. *Journal of Clinical Investigation*.

[76] Huang Y., Cohen J. C., Hobbs H. H. (2011). Expression and Characterization of a PNPLA3 Protein Isoform (I148M) Associated With Nonalcoholic Fatty Liver Disease. *Journal of Biological Chemistry*.

[77] Basantani M. K., Sitnick M. T., Cai L. (2011). Pnpla3/Adiponutrin Deficiency in Mice Does Not Contribute to Fatty Liver Disease or Metabolic Syndrome. *Journal of Lipid Research*.

[78] BasuRay S., Wang Y., Smagris E., Cohen J. C., Hobbs H. H. (2019). Accumulation of PNPLA3 on Lipid Droplets Is the Basis of Associated Hepatic Steatosis. *Proceedings of the National Academy of Sciences of the United States of America*.

[79] Yang A., Mottillo E. P., Mladenovic-Lucas L., Zhou L., Granneman J. G. (2019). Dynamic Interactions of ABHD5 With PNPLA3 Regulate Triacylglycerol Metabolism in Brown Adipocytes. *Nature Metabolism*.

[80] Lindén D., Ahnmark A., Pingitore P. (2019). Pnpla3 Silencing With Antisense Oligonucleotides Ameliorates Nonalcoholic Steatohepatitis and Fibrosis in *Pnpla3* I148M Knock-in Mice. *Molecular Metabolism*.

[81] Armisen J., Rauschecker M., Sarv J. (2025). AZD2693, a PNPLA3 Antisense Oligonucleotide, for the Treatment of MASH in 148M Homozygous Participants: Two Randomized Phase I Trials. *Journal of Hepatology*.

